# Factors Associated with Healthcare Professionals’ Attitudes Toward Migrant Patients: A Cross-Sectional, Exploratory Study

**DOI:** 10.3390/healthcare14182984

**Published:** 2026-09-12

**Authors:** Zülal Soylu Karaca, Miray Akkuş, Hande Başak

**Affiliations:** 1 Department of Midwifery, Faculty of Health Sciences, Istanbul Topkapı University, 34087 Istanbul, Turkey; zulalsoylu@topkapi.edu.tr; 2 Department of Obstetrics and Gynecology Nursing, Institute of Health Sciences, Dokuz Eylül University, 35330 Izmir, Turkey; 3 Department of Obstetrics and Gynecology Nursing, Faculty of Nursing, Dokuz Eylül University, 35330 Izmir, Turkey; hande.yagcan@gmail.com

**Keywords:** migrants, cultural and linguistic diversity, health services, culturally sensitive care

## Abstract

**Objective**: To determine healthcare professionals’ attitudes toward migrant patients and explore the sociodemographic and migration-related factors associated with these attitudes. **Methods**: This descriptive, cross-sectional, exploratory study was conducted with 390 healthcare professionals reached through a Facebook-based professional community and subsequently via other social media platforms using snowball sampling. Data were collected using a Demographic Information Form and the Healthcare Professionals’ Attitudes Toward Migrants Scale. Data Analysis involved the use of independent-samples *t*-tests or one-way ANOVAs, or Mann–Whitney U or Kruskal–Wallis tests, based on distribution characteristics; effect sizes and 95% confidence intervals were calculated for each comparison, and the Benjamini–Hochberg false discovery rate (FDR) correction was applied to all 96 comparisons reported. Additionally, a multiple linear regression analysis was performed using conceptually justified predictors to identify independent and interrelated factors. **Results**: The mean total attitude score was 38.47 ± 10.71 (the total score was calculated by reversing the scores on the positive attitude subscale; thus, a higher score indicates a more negative overall attitude; see Methods). In pairwise comparisons, attitude scores differed according to marital status, job satisfaction, the number of migrant patients encountered, language barriers, cultural differences, access to interpreters, perceived competence in providing care, perceptions of migrants’ attitudes, perceived social prejudice, workload, and willingness to receive training on migrant health (raw *p* < 0.05 in 42 of 96 comparisons; 39 after FDR correction; 22 of these 42 also met a stricter α = 0.001 threshold applied given the large number of repeated comparisons). Multivariable regression (R^2^ = 0.398) showed that perceived social prejudice (standardized β = 0.300), reluctance to pursue education (β = 0.287), and increased workload (β = 0.181) were independently associated with higher total attitude scores. Inaccessibility of interpreters (*p* = 0.010) and negative perception of migrants’ attitudes (*p* = 0.029) showed associations at the conventional *p* < 0.05 level but did not meet the stricter α = 0.001 threshold. **Conclusions**: In this exploratory sample, healthcare professionals’ attitudes toward migrant patients were associated not only with individual characteristics but also with communication barriers, cultural competence, and working conditions. Given that the study design was cross-sectional and based on non-probability sampling, these associations should be interpreted with caution and not assumed to be causal. Strengthening professional interpreter services, expanding cultural competence training, and evaluating institutional support strategies through future controlled or longitudinal studies may help improve healthcare professionals’ attitudes and promote migrant-friendly, equitable healthcare services.

## 1. Introduction

According to the Dictionary of International Migration Terms, migration is defined as the movement of a person or group of people across an international border or within a state. This definition encompasses refugees, displaced persons, economic migrants, and individuals who relocate for reasons such as family reunification [1]. In recent decades, international migration has increased significantly and has become a global phenomenon with major implications for health systems worldwide. Migrants face numerous barriers to accessing health care in host countries, including language differences, cultural alienation, socioeconomic disadvantage, and discrimination [2,3]. These barriers contribute to unequal access to health care and can further exacerbate health inequities, particularly among vulnerable groups such as women, children, older adults, and individuals with chronic conditions [4,5]. Health care professionals play a critical role in ensuring that health care services are accessible, inclusive, and safe for migrants. Healthcare professionals’ attitudes toward migrants can directly influence the quality of care provided, patient-healthcare professional communication, satisfaction with services, and treatment adherence [6,7]. While positive and inclusive attitudes form the foundation of culturally sensitive care, negative attitudes can lead migrants to avoid or delay seeking health care due to prejudice and stigmatization [8].

The literature indicates that healthcare professionals’ attitudes toward migrants are influenced by various factors at the individual, organizational, and societal levels. Professional experience, educational level, knowledge of cross-cultural care, and prior experience working with migrants are among the primary individual factors shaping these attitudes [8,9]. Furthermore, heavy workloads, time constraints, language barriers, and a lack of institutional support can increase burnout among healthcare professionals, which in turn can foster negative attitudes toward migrants [10].

The discrimination and stigmatization that migrants experience within the health care system are not limited to individual attitudes. Still, they are also linked to the structural characteristics of the health care system. Negative attitudes among healthcare professionals can lead to outcomes such as care inequalities, communication breakdowns, and reduced patient safety [11]. Therefore, identifying healthcare professionals’ attitudes toward migrants is considered a useful step that can contribute to the development of cross-cultural care.

Due to its geographic location, Turkey has long been among the countries receiving high levels of migration and provides health services to migrant groups with diverse cultural backgrounds. In this context, examining healthcare professionals’ attitudes toward migrants can help clarify the current situation and inform the development of migrant-friendly health service models [12].

This study aimed to examine healthcare professionals’ attitudes toward migrants and their associations with sociodemographic, professional, and migrant care-related characteristics.

## 2. Materials and Methods

This cross-sectional study is reported in accordance with the STROBE (Strengthening the Reporting of Observational Studies in Epidemiology) statement for cross-sectional studies [13]; a completed STROBE checklist, including the items on selection bias, exclusion criteria, and participant flow addressed below, is provided as Supplementary Material.

### 2.1. Study Type and Sample

**Figure 1:**
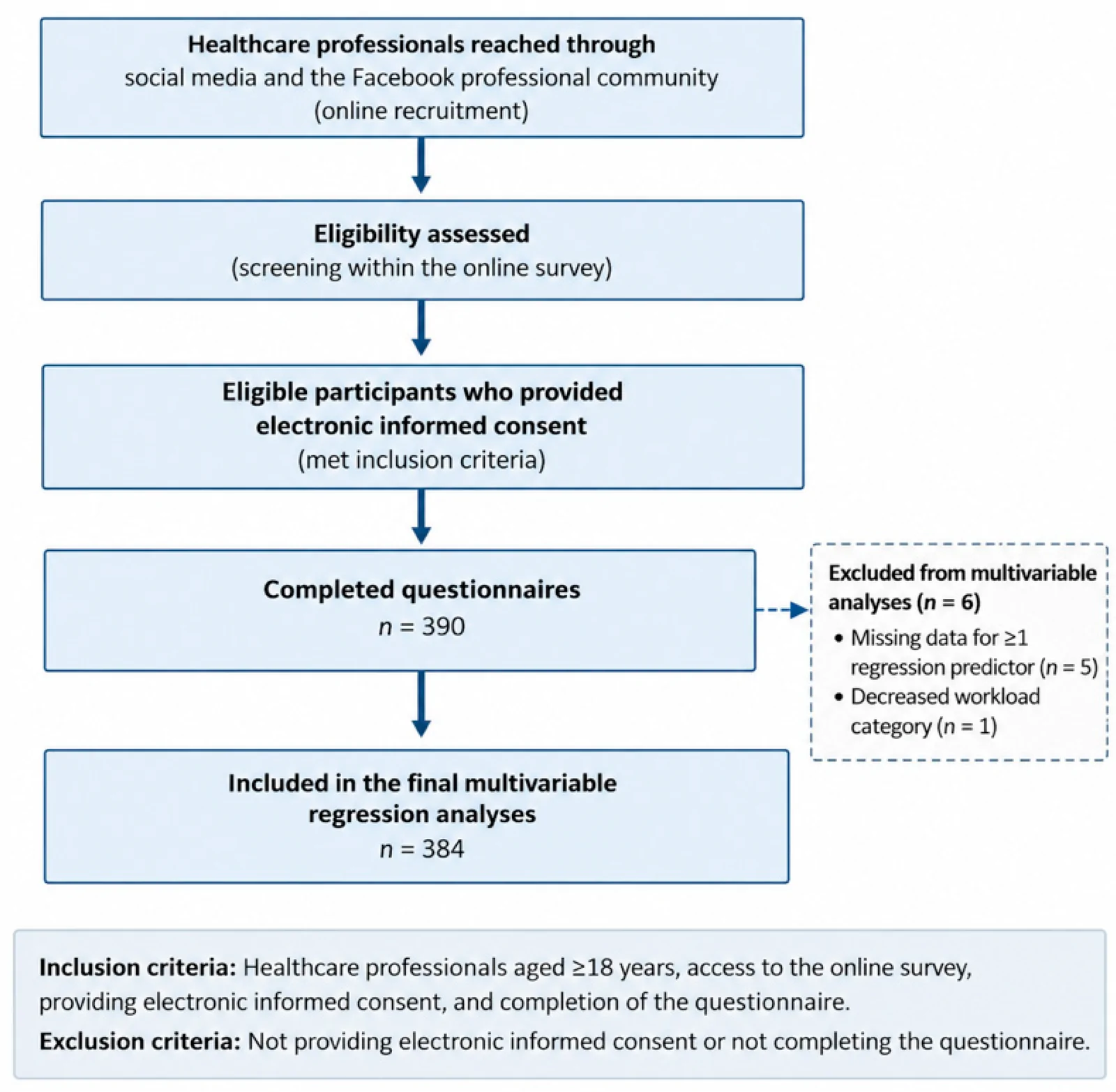
Flowchart of participant recruitment and inclusion.

This study was designed as a descriptive, cross-sectional study to examine the attitudes of healthcare professionals toward migrant patients and the factors associated with these attitudes in a convenience sample. The study population consisted of healthcare professionals who were members of the Facebook Reproductive Health Volunteers Community, which had approximately 15,533 members at the time of data collection. Given the known population size, a finite population sample size calculation based on a 95% confidence level and a 5% margin of error, using the standard finite-population correction formula [14], indicated a minimum required sample size of 375 participants. In addition, an a priori power analysis was conducted using G*Power 3.1.9.7 [15] for an omnibus F test in linear multiple regression (fixed model, R^2^ deviation from zero), assuming 12 predictors, an effect size of f^2^ = 0.15, α = 0.05, and 95% statistical power. This analysis indicated a minimum required sample size of 184 participants. Tests of individual regression coefficients were two-sided. The effect size of f^2^ = 0.15 was selected based on the conventional medium-effect benchmark for multiple regression [16]. Recruitment continued until the number of completed, eligible questionnaires exceeded both the finite population-based and regression-based minimum requirements; the achieved sample size is reported in Section 3 (Findings) and in the participant flow diagram (Figure 1).

Participants were eligible if they were healthcare professionals aged ≥18 years, had access to the online survey, provided electronic informed consent, and completed the questionnaire. Individuals who did not provide electronic informed consent or did not complete the questionnaire were excluded from the final analytical sample.

### 2.2. Data Collection

Participants were initially recruited through the Facebook Reproductive Health Volunteers Community. Data were collected between March 2025 and March 2026. All questionnaire items were set as mandatory in the online survey; therefore, participants could submit the questionnaire only after completing all required items, and no incomplete questionnaires were included in the Analysis. Only completed questionnaires were included in the final dataset; the number analyzed is reported in Figure 1. The number of individuals who accessed the survey link without submitting a response was not available; therefore, a response rate could not be calculated. To prevent duplicate submissions, e-mail verification was required, and the survey was configured to allow only one response per verified account. The survey link was shared on social media, and participants were encouraged to share it with other eligible healthcare professionals, resulting in snowball sampling across additional platforms (Facebook, Instagram, Twitter, and others). Inclusion criteria were as follows: (1) being a healthcare professional, (2) being a member of the Facebook Reproductive Health Volunteers Community or having accessed the survey through the community’s posts, (3) being 18 years of age or older, (4) providing electronic informed consent, and (5) completing the online survey. Data were collected using the Individual Assessment Form and the Health Professionals’ Attitudes Toward Migrants Scale, administered via Google Forms. Participants’ geographical location was not collected as a study variable; therefore, the sample’s geographical distribution could not be determined.

Individual Assessment Form: Developed by the researchers in line with the literature, this form comprises 24 questions on participants’ sociodemographic characteristics and work lives [17,18].

Health Professionals’ Attitudes Toward Migrants Scale: Developed by Karagöz and Karaşin, this 5-point Likert-type measure comprises 3 factors and 14 items (1: Strongly Disagree, 2: Disagree, 3: Neither Agree Nor Disagree, 4: Agree, 5: Strongly Agree) [18]. The scale consists of three subscales: Items 1–7 represent the negative attitude subscale, items 8–10 represent the inhibitory attitude subscale, and items 11–14 represent the positive attitude subscale. Subscale scores are calculated by directly summing the relevant items (there are no reverse-scored items at the subscale level); higher scores on the negative and inhibitory attitude subscales indicate more negative/restrictive attitudes, while a higher score on the positive attitude subscale indicates a more positive attitude. For the total scale score only, in accordance with the scoring rule specified by the scale developers [19], items on the positive attitude subscale are reverse-scored (recoded value = 6 − original item response) before being summed with the negative attitude and inhibitory attitude subscales. Under this rule, the total score ranges from 14 to 70; unlike the positive subscale, which, when considered on its own, can indicate a more positive attitude toward migrants, a high total score indicates a more negative overall attitude toward migrants.

The reliability coefficient for the entire scale (Cronbach’s α, calculated under this scoring rule) is 0.875, as reported by the scale developers; in the current sample, it was 0.867. Cronbach’s α for the subscales is 0.855 for the negative attitude subfactor (0.890 in the current sample), 0.752 for the inhibitory attitude subfactor (0.802 in the current sample), and 0.700 for the positive attitude subfactor (0.780 in the current sample) [19].

### 2.3. Data Analysis

The data were analyzed using IBM SPSS Statistics 27 and Python (version 3.14.2; pandas, SciPy, and statsmodels). Descriptive statistics are presented as frequency, percentage, mean, standard deviation, median, and interquartile range (Q1–Q3: first quartile [25th percentile]—third quartile [75th percentile]). The data’s conformity to a normal distribution, as well as its skewness and kurtosis, were examined using the Shapiro–Wilk test and Q–Q plots. This evaluation revealed that the negative attitude subscale (skewness = −0.549; kurtosis = −0.710) and the total scale score (skewness = 0.231; kurtosis = −0.130) showed an approximately normal distribution, whereas the inhibitory attitude (skewness = 1.553; kurtosis = 1.858) and positive attitude (skewness = −1.704; kurtosis = 1.246) subscales exhibited a markedly skewed distribution. Therefore, parametric tests—independent-samples *t*-tests for two-group comparisons and one-way ANOVA for comparisons involving three or more groups—were used for the negative-attitude and total scores. Nonparametric tests—the Mann–Whitney U test for two-group comparisons and the Kruskal–Wallis H test for comparisons involving three or more groups—were used for the inhibitory- and positive-attitude subscale scores.

Descriptive statistics for each comparison were reported as both mean ± standard deviation and median (Q1–Q3). For comparisons involving nonparametric tests, medians and interquartile ranges were considered more appropriate for interpretation.

Effect sizes and 95% confidence intervals: An appropriate effect size was calculated for each comparison: Cohen’s d for two-group parametric comparisons, eta-squared (η^2^) for multi-group parametric comparisons (ANOVA), rank-biserial correlation (r) for two-group nonparametric comparisons, and epsilon-squared (ε^2^) for multi-group nonparametric comparisons (Kruskal–Wallis). The 95% confidence intervals for all effect sizes were calculated using bootstrap resampling with 3000 repetitions (percentile method).

Multiple-test correction: A total of 96 comparisons were conducted between the four attitude outcomes (negative, inhibitory, positive, and total) and 24 participant- and care-related characteristics. To control the false discovery rate across multiple comparisons, the Benjamini–Hochberg false discovery rate (FDR) correction was applied to all 96 *p*-values, and an adjusted q-value was calculated for each comparison (α = 0.05). Bonferroni post hoc comparisons were additionally performed following significant omnibus tests.

Multivariable Analysis: A multiple linear regression was conducted using conceptually grounded predictors. The total attitude score was specified as the primary outcome, while the negative attitude subscale was examined in a sensitivity Analysis. The predictors included marital status, job satisfaction, language barriers, cultural differences, lack of access to an interpreter, perceived low effectiveness in providing care, negative perceptions of migrants’ attitudes, perceived social prejudice, increased workload, and reluctance to receive further education. The “decreased workload” category (*n* = 1) was excluded from the regression Analysis. The number of migrant patients encountered in the previous month was not included because the corresponding free-text/numeric data could not be reliably parsed and did not reproduce the reported categorical distribution. Similarly, the most frequently provided health service was not included because the free-text responses resulted in numerous sparse categories. Multicollinearity was assessed using variance inflation factors (VIFs), all of which were below 1.25.

Handling of small subgroups: When group categories contained very few participants (e.g., n ≤ 5), the results were presented descriptively and interpreted with caution. When such categories were included in a broader inferential comparison, this was indicated in the relevant table footnote. For the workload variable, the analysis was repeated after excluding the “decreased workload” category because it contained only one participant (*n* = 1).

Distinction between primary and exploratory Analysis: Based on the study aims and conceptual relevance, Analysis focusing on language barriers, cultural differences, interpreter access, perceived effectiveness of care, perceived attitudes of migrants, perceived social prejudice, changes in workload, willingness to receive further education, marital status, and job satisfaction were considered the primary Analysis. These variables were also included in the multivariable regression models. The remaining sociodemographic and professional characteristics (age, gender, education level, occupation, professional experience, voluntary choice of occupation, and perceived suitability of the occupation), as well as the migrant care-related characteristics (number of migrant patients encountered, most frequently encountered migrant group, age group of migrant patients, and type of healthcare service provided), were considered exploratory Analysis and were interpreted accordingly.

### 2.4. Ethical Considerations

Before data collection, ethical approval for the study was obtained from the Dokuz Eylül University Ethics Committee for Non-Interventional Research (Decision No.: 2025/11-09, Date: 26 March 2025, File No.: 9617-GOA). Permission to use the Health Professionals’ Attitudes Toward Migrants Scale was obtained from the scale developers before the study began. Participation in the study was entirely voluntary. Before accessing the online survey, participants were provided with detailed information regarding the study’s objectives, procedures, data confidentiality, and their right to withdraw from the study at any time without any consequences. Electronic informed consent was obtained from all participants before they completed the survey. The study was conducted in accordance with the ethical principles of the Declaration of Helsinki. No personally identifiable information was collected; all data were anonymized and stored securely. The collected data were used solely for scientific research purposes.

### 2.5. Potential Sources of Bias

Several potential sources of bias were considered. Selection bias may have occurred because participants were recruited via convenience and snowball sampling through social media, which may have preferentially reached healthcare professionals more interested in migrant or reproductive health. The sample was also predominantly composed of nurses/midwives and women, which may limit the generalizability of the findings to other healthcare professional groups. To reduce duplicate participation, email verification and a one-response-per-verified-account procedure were used. All questionnaire items were mandatory, which minimized item-level missing data; however, the number of individuals who accessed the survey but did not complete it was unavailable, so a response rate could not be calculated. Because the data were collected using self-report measures, social desirability and common method biases cannot be ruled out. Geographic information was not collected, preventing assessment of regional variation.

## 3. Findings

A total of 390 healthcare professionals completed the online questionnaire and were included in the final Analysis.

The sociodemographic characteristics of the participants are presented in Table 1. A total of 390 healthcare professionals participated in the study. The mean age was 31.33 ± 7.72 years (median: 29 years; range: 22–60 years). The majority of participants were women (90.3%), nurses or midwives (88.7%), and college graduates (68.5%). More than one-third of the participants (36.7%) had 10 years or more of professional experience. Approximately half of the participants reported choosing their profession voluntarily (50.3%), while 62.8% felt their profession was a good fit for them. In terms of job satisfaction, 42.8% reported being somewhat satisfied with their profession, while 37.2% reported being satisfied (Table 1).

Table 2 summarizes characteristics related to the care of migrant patients. Approximately two-fifths (39.2%) of participants reported seeing three or fewer migrant patients in the past month, while 25.4% reported seeing 16 or more migrant patients. The most frequently encountered migrant group was women (36.2%), followed by newborns/children (21.8%). While the majority of healthcare professionals reported providing outpatient or emergency care (31.3%), 23.1% indicated that they provided services across all primary care settings. Overall, 62.3% of participants reported encountering barriers when providing care to migrant patients, while 26.4% reported encountering them to some extent (346 participants—88.7% of the sample—reported either full or partial barriers). Since the question regarding the type of barrier was answered only by these 346 participants, the percentages for barrier types were calculated using the correct denominator of *n* = 346 (participants who reported any barrier) rather than the total sample of *n* = 390: 90.2% reported a language barrier, 51.4% reported cultural differences, and 39.9% reported insufficient medical history information as the type of barrier. More than half of the participants (53.6%) reported that interpreters were available when needed, while 51.8% perceived themselves as only partially effective in providing care to migrant patients. Most participants perceived migrant patients’ attitudes toward health professionals as neutral (63.1%), and 57.7% believed that migrants’ attitudes toward health and illness made the delivery of health care more difficult. In addition, 69.2% reported that providing care to migrant patients increases their workload. While only 3.3% had received in-service training on migrant health, 48.2% were willing to receive such training (Table 2).

The descriptive statistics, internal consistency coefficients, and distribution characteristics of the Health Professionals’ Attitudes Toward Migrants Scale are presented in Table 3. The mean total scale score was 38.47 ± 10.71, with mean subscale scores of 25.92 ± 7.39 for negative attitudes, 5.40 ± 3.06 for inhibitory attitudes, and 16.85 ± 3.48 for positive attitudes. As explained in detail in Section 2.2, the total score is calculated by reversing the scoring of the positive attitude items; therefore, the total score is not the arithmetic sum of the three subscale means reported in their original directions.

The internal consistency of the total scale is at an acceptable level (Cronbach’s α = 0.867); the α coefficients for the subscales are also at acceptable levels (ranging from 0.780 to 0.890). The skewness and kurtosis values for all subscales and the total score are within acceptable ranges.

Overall, attitude scores did not differ significantly by gender, occupation, years of professional experience, whether the occupation was chosen voluntarily, or perceived suitability of the occupation (all *p* > 0.05). Married participants showed higher scores on the negative attitude, inhibitory attitude, and total attitude scales compared with single participants; however, these associations did not meet the stricter α = 0.001 threshold (*p* = 0.007, *p* = 0.016, and *p* = 0.005, respectively). Educational level showed an association with inhibitory attitude scores at the conventional *p* < 0.05 level (*p* = 0.028); however, this association did not meet the stricter α = 0.001 threshold. The post hoc comparisons showed that high school graduates scored higher than undergraduate and graduate degree holders. In addition, participants who were dissatisfied with their profession had higher negative attitude and total attitude scores compared with those who were satisfied; however, these associations did not meet the stricter α = 0.001 threshold (*p* = 0.018 and *p* = 0.016, respectively) (Table 4).

Comparisons of attitude scale scores according to migrant patient and care characteristics are presented in Table 5. The number of migrant patients encountered in the past month was associated with negative, positive, and total attitude scores at the conventional *p* < 0.05 level; however, none of these associations met the stricter α = 0.001 threshold (*p* = 0.002, 0.042, and 0.012, respectively). A Bonferroni post hoc analysis showed that healthcare professionals who encountered 16 or more migrant patients per month had higher negative attitude and total attitude scores compared with those who encountered three or fewer migrant patients; however, these pairwise differences did not meet the stricter α = 0.001 threshold. No statistically significant differences were observed in attitude scale or subscale scores across the most frequently encountered migrant groups (all *p* > 0.05). The age group of the most frequently encountered migrants was associated with negative attitude scores at the conventional *p* < 0.05 level (*p* = 0.015), but this association did not meet the stricter α = 0.001 threshold. The association with total attitude scale scores (*p* = 0.050) did not meet either the conventional *p* < 0.05 or the stricter α = 0.001 threshold.

The most frequently provided type of health service was associated with negative attitude (*p* = 0.015), inhibitory attitude (*p* = 0.006), and total attitude scale scores (*p* = 0.003) at the conventional *p* < 0.05 level; however, none of these associations met the stricter α = 0.001 threshold. Post hoc Analysis showed that providers of child health monitoring services and those providing all primary care services had higher negative attitude and total attitude scores compared with the “other services” category; however, these pairwise differences should be interpreted cautiously and were not considered statistically significant under the stricter α = 0.001 threshold. In this table, categories with very small sample sizes (migrants aged 11–15, *n* = 2; reproductive health services, *n* = 3; services for sexually transmitted infections, *n* = 4) were retained for completeness but should not be interpreted as reliable group comparisons (Table 5).

**Table 5:** Comparison of Attitude Scale Scores According to Migrant Patient and Care Characteristics. (All comparisons in this table are secondary/exploratory in nature; they were not included in the multivariate model—see Section 2.3).

Variable	Category	Negative Attitude Mean ± SD	Inhibitory Attitude Mean ± SD	Positive Attitude Mean ± SD	Total Score Mean ± SD
Number of migrant patients seen last month	≤3 (1)	24.82 ± 7.00	5.15 ± 3.09	17.04 ± 3.18	36.93 ± 11.13
	4–9 (2)	26.22 ± 7.49	5.03 ± 2.50	17.61 ± 2.70	37.64 ± 9.54
	10–15 (3)	24.83 ± 8.23	5.75 ± 3.40	16.01 ± 4.38	38.56 ± 10.47
	≥16 (4)	28.19 ± 6.81	5.80 ± 3.09	16.63 ± 3.59	41.36 ± 10.53
	F/H	4.957	1.530	2.754	3.675
	*p*	**0.002** (post hoc: 4 > 1, 4 > 3)	0.206	**0.042** (post hoc: 2 > 3)	**0.012** (post hoc: 4 > 1)
Most common migrant group	Newborns/Children	26.68 ± 7.72	5.51 ± 2.95	16.33 ± 3.69	39.86 ± 10.78
	Female	25.70 ± 7.23	5.57 ± 3.40	16.89 ± 3.27	38.38 ± 10.56
	Male	23.96 ± 7.15	5.21 ± 3.21	17.17 ± 3.77	36.00 ± 11.57
	Elderly	24.00 ± 6.67	3.93 ± 1.33	17.50 ± 2.21	34.43 ± 8.18
	All groups	26.89 ± 7.40	5.38 ± 2.72	16.97 ± 3.59	39.30 ± 10.55
	F/H	1.845	0.997	0.739	1.707
	*p*	0.119	0.409	0.566	0.148
The most common age group among migrants	0–5 years (1)	26.96 ± 7.46	5.55 ± 2.91	16.20 ± 3.65	40.31 ± 10.27
	Ages 6–10 (2)	25.00 ± 8.92	4.88 ± 2.68	17.63 ± 3.37	36.25 ± 13.20
	Ages 11–15 (3)	20.50 ± 13.44	5.00 ± 2.83	19.00 ± 1.41	30.50 ± 17.68
	Ages 16–20 (4)	31.77 ± 4.85	5.62 ± 3.66	16.85 ± 3.56	44.54 ± 9.03
	≥21 years (5)	25.45 ± 7.17	5.40 ± 3.13	16.95 ± 3.44	37.90 ± 10.53
	F/H	3.140	0.247	1.245	2.398
	*p*	**0.015** (post hoc: 4 > 5)	0.912	0.291	0.050
Most frequently provided healthcare service	Outpatient care/Emergency care (1)	25.61 ± 7.13	5.39 ± 3.13	17.00 ± 3.44	38.00 ± 10.97
	Vaccination (2)	24.08 ± 7.14	4.38 ± 2.75	16.23 ± 4.90	36.23 ± 7.38
	Prenatal and postnatal care (3)	25.62 ± 7.59	5.43 ± 3.23	16.56 ± 3.54	38.49 ± 10.80
	Child health monitoring (4)	29.06 ± 6.71	7.29 ± 3.60	15.90 ± 3.95	44.45 ± 10.87
	Reproductive health (5) *	23.33 ± 7.23	3.00 ± 0.00	16.33 ± 3.21	34.00 ± 7.00
	Services for STIs (6) *	24.75 ± 4.79	4.75 ± 2.06	17.75 ± 2.63	35.75 ± 6.90
	All services (7)	27.36 ± 7.18	5.47 ± 2.98	16.87 ± 3.49	39.96 ± 10.64
	Other (8)	23.13 ± 7.88	4.48 ± 1.91	17.67 ± 2.68	33.93 ± 9.16
	F/H	2.526	2.874	0.915	3151
	*p*	**0.015** (post hoc: 4 > 8, 7 > 8)	**0.006** (post hoc: 4 > 8)	0.495	**0.003** (post hoc: 4 > 8, 7 > 8)

Data are presented as mean ± SD. One-way ANOVA (F) or the Kruskal–Wallis (H) test was used as appropriate. A Bonferroni post hoc analysis was applied following overall comparisons with *p* ≤ 0.001. Statistically significant *p*-values (*p* ≤ 0.001) are shown in bold. * Indicates the category with the smallest sample size.

Comparisons of attitude scale scores regarding barriers, perceptions, workload, and education related to the care of migrant patients are presented in Table 6. Healthcare professionals who reported encountering barriers when providing care to migrant patients had significantly higher negative attitude scores (*p* < 0.001) and total attitude scale scores (*p* < 0.001) than those who did not report such barriers. Participants who reported cultural differences exhibited significantly higher negative attitude scores (*p* < 0.001), whereas the association for reported language barriers did not meet the stricter α = 0.001 threshold (*p* = 0.017). Insufficient knowledge of medical history was not found to be significantly associated with any attitude scale or subscale score (all *p* > 0.05). Migrant patients’ perceived attitudes toward healthcare professionals were significantly associated with negative attitude scores (*p* < 0.001), positive attitude scores (*p* = 0.001), and total attitude scores (*p* < 0.001). The association with inhibitory attitude scores (*p* = 0.003) did not meet the stricter α = 0.001 threshold. Participants who believed that social prejudice plays a role in care provision had significantly higher scores on negative attitude, inhibitory attitude, and total attitude scales—but lower scores on positive attitude—compared to those who did not (all *p* < 0.001). Participants who perceived that providing care to migrant patients increased their workload showed significantly higher negative attitude scores and total attitude scores than those who reported no change in workload (both *p* < 0.001). The differences in inhibitory and positive attitude scores did not meet the stricter α = 0.001 threshold (*p* = 0.003 and *p* = 0.032, respectively). The two-group comparison remained highly significant for all outcomes (negative attitude: t = −6.981, *p* < 0.001; inhibitory attitude: t = −3.044, *p* = 0.003; positive attitude: t = 2.155, *p* = 0.032; total score: t = −6.405, *p* < 0.001), which is consistent with the direction of the original finding. Having received in-service training on migrant health was not associated with attitude scale scores (all *p* > 0.05); we interpret this finding carefully below. However, the desire to receive such training was found to be significantly associated with all dimensions of the attitude scale (all *p* < 0.001); participants who were not willing to receive training exhibited higher negative, inhibitory, and total attitude scores, while those willing to receive training exhibited higher positive attitude scores (Table 6).

### 3.1. Effect Sizes, 95% Confidence Intervals, and Multiple Comparison Correction

For all 96 comparisons reported in Table 4, Table 5 and Table 6, the median (Q1–Q3) values, appropriate effect size measures (Cohen’s d, η^2^, rank-biserial r, or ε^2^) and their bootstrap 95% confidence intervals, along with q-values following Benjamini–Hochberg FDR correction, are presented in full in a separate supplementary table (Supplementary Table S1) due to length constraints. In summary: 42 of the 96 comparisons met the uncorrected *p* < 0.05 threshold; after FDR correction, this number decreased to 39. The three comparisons that lost their significance after FDR correction were: the association between changes in workload and positive attitude (*p* = 0.043; q = 0.099), the association between the age group of migrants and the total score (*p* = 0.038; q = 0.089—this had already been reframed as a non-significant trend in Section 3), and the association between the impact of care and inhibitory attitudes (*p* = 0.034; q = 0.082). Among the findings that retained their significance even after FDR correction, those with the largest effect sizes—reluctance to pursue education Cohen’s d = 0.914 (95% CI: 0.710–1.140) for the total score, perceived social prejudice (moderate-to-large effect sizes) and the experience of encountering barriers—can be considered the most robust findings of this study; in contrast, comparisons with *p*-values close to 0.05 and/or small effect sizes (e.g., various sociodemographic variables) should be interpreted with caution. In response to reviewer feedback regarding the large number of repeated pairwise comparisons in Table 4, Table 5 and Table 6, we additionally applied a more conservative significance threshold of α = 0.001. Of the 42 comparisons that met the conventional *p* < 0.05 criterion, 22 (52%) remained significant at this stricter threshold; all 22 also survived Benjamini–Hochberg FDR correction. These 22 comparisons involved encountering barriers while providing care, cultural differences, availability of an interpreter, perceived effectiveness of care, migrant patients’ perceived attitudes toward healthcare professionals, perceived social prejudice, the perceived impact of migrants’ attitudes on care delivery, changes in workload, and willingness to receive further training—the same variables that were retained as predictors in the multivariable models (Table 7 and Table 8) and that showed the largest effect sizes. Findings that were significant only at the conventional *p* < 0.05 level (e.g., several sociodemographic comparisons in Table 4) should be regarded as more tentative.

### 3.2. Multivariate Analysis

Multiple linear regression Analysis was conducted to determine which factors were independently associated with the total attitude score (*n* = 384, after listwise deletion). The model is statistically significant (F(10, 373) = 23.28; *p* < 0.001) and explains 39.8% of the variance in the total score (R^2^ = 0.398; adjusted R^2^ = 0.382). All variance inflation factors were below 1.25, indicating no multicollinearity issues (Table 7). Following reviewer feedback, we additionally re-estimated the total-score model with the type of healthcare professional (occupation: physician, nurse/midwife [reference], other) added as a covariate; this sensitivity Analysis is reported in Section 3.3 and Table 9.

When all other predictors were held constant, perceived social prejudice (B = 6.50; 95% CI 4.71–8.30; *p* < 0.001; standardized β = 0.300), increased workload (B = 4.22; 95% CI 2.15–6.28; *p* < 0.001; β = 0.181), and reluctance to pursue education (B = 6.15; 95% CI 4.31–7.99; *p* < 0.001; β = 0.287) were independently associated with higher total attitude scores. Inaccessibility of an interpreter (*p* = 0.010) and negative perception of migrants’ attitudes (*p* = 0.029) showed positive associations at the conventional *p* < 0.05 level but did not meet the stricter α = 0.001 threshold. Marital status, job dissatisfaction, reporting a language barrier, reporting cultural differences, and perceived low effectiveness were not independently associated with the total score. This suggests that the bivariate associations observed in Table 4 and Table 6 may be explained, at least partly, by confounding or shared variance with other variables included in the multivariable model.

As a sensitivity Analysis, the same model was repeated using the negative attitude subscale (the component of the total score that is “conceptually the most clearly interpretable”; see Limitations), and consistent results were obtained (R^2^ = 0.327; adjusted R^2^ = 0.309; F(10, 373) = 20.81; *p* < 0.001): perceived social prejudice (B = 3.72; 95% CI 2.38–5.05; *p* < 0.001; β = 0.250), increased workload (B = 3.43; 95% CI 1.94–4.93; *p* < 0.001; β = 0.216) and reluctance to pursue education (B = 3.66; 95% CI 2.31–5.01; *p* < 0.001; β = 0.249) remained the strongest independent predictors in this model as well; reporting cultural differences (*p* = 0.057) and negative perception of migrants’ attitudes (*p* = 0.055) were not statistically significant (Table 8). The consistency of the two models supports our interpretation that these three factors (perceived social prejudice, increased workload, and reluctance to pursue education) are the most robust independent predictors in this sample (remaining significant in both unstandardized and standardized Analysis, as well as after FDR correction).

### 3.3. Sensitivity Analysis: Type of Healthcare Professional

In response to reviewer feedback, the total-score multivariable model reported in Table 7 was re-estimated with the type of healthcare professional (occupation) added as two additional predictors—physician and “other” allied health titles, each compared against the reference category of nurse/midwife (*n* = 384, after listwise deletion; model F(12, 371) = 19.31, *p* < 0.001; R^2^ = 0.399, adjusted R^2^ = 0.379). Adding occupation did not materially change the model: neither the physician category (B = −1.828, 95% CI −10.562 to 6.907, *p* = 0.682) nor the “other” category (B = −0.497, 95% CI −3.639 to 2.645, *p* = 0.757) was independently associated with the total attitude score, and the pattern, magnitude, and significance of all other predictors were essentially unchanged from Table 7 (Table 9). Adjustment for occupation did not materially alter the estimated associations reported in Table 7.

**Table 9:** Sensitivity Analysis: Multivariate Linear Regression Model for the Total Attitude Score with Type of Healthcare Professional Added (*n* = 384).

Predictor	B (Unstandardized)	95% CI	Standardized β
**Constant (Cutoff point)**	**25.621**	**22.702–28.539**	**—**
Marital status: Married (ref: Single)	0.992	−0.791–2.775	0.046
Job dissatisfaction (ordinal: Yes = 0, Partially = 1, No = 2)	0.107	−1.135–1.349	0.007
Reporting a language barrier (ref: Did not report)	−0.875	−3.274–1.524	−0.032
Reporting cultural differences (ref: Those who did not report)	1.242	−0.497–2.980	0.058
**Inaccessibility of an interpreter (ref: Accessible)**	**2.430**	**0.617–4.242**	**0.113**
Perceived low effectiveness (ordinal: Yes = 0, Partially = 1, No = 2)	1.204	−0.243–2.652	0.074
**Negative perception of migrants’ attitudes (ordinal: Positive = 0, Neutral = 1, Negative = 2)**	**1.910**	**0.173–3.648**	**0.105**
**Perception of social prejudice (ref: Those who do not perceive it)**	**6.481**	**4.643–8.319**	**0.299**
**Increase in workload (ref: No change; *n* = 1—the “decreased” category excluded)**	**4.224**	**2.157–6.291**	**0.182**
**Reluctance to pursue education (ref: Willing)**	**6.216**	**4.354–8.077**	**0.290**
Type of healthcare professional: Physician (ref: Nurse/Midwife)	−1.828	−10.562–6.907	−0.023
Type of healthcare professional: Other (ref: Nurse/Midwife)	−0.497	−3.639–2.645	−0.014

B: unstandardized regression coefficient; CI: confidence interval; β: standardized regression coefficient. Calculated using Robust (HC3) standard errors. R^2^ = 0.399; adjusted R^2^ = 0.379; F(12, 371) = 19.31; *p* < 0.001. Statistically significant predictors (*p* < 0.001) are shown in bold.

### 3.4. Exploratory Pathway (Mediation) Analysis

**Figure 2:**
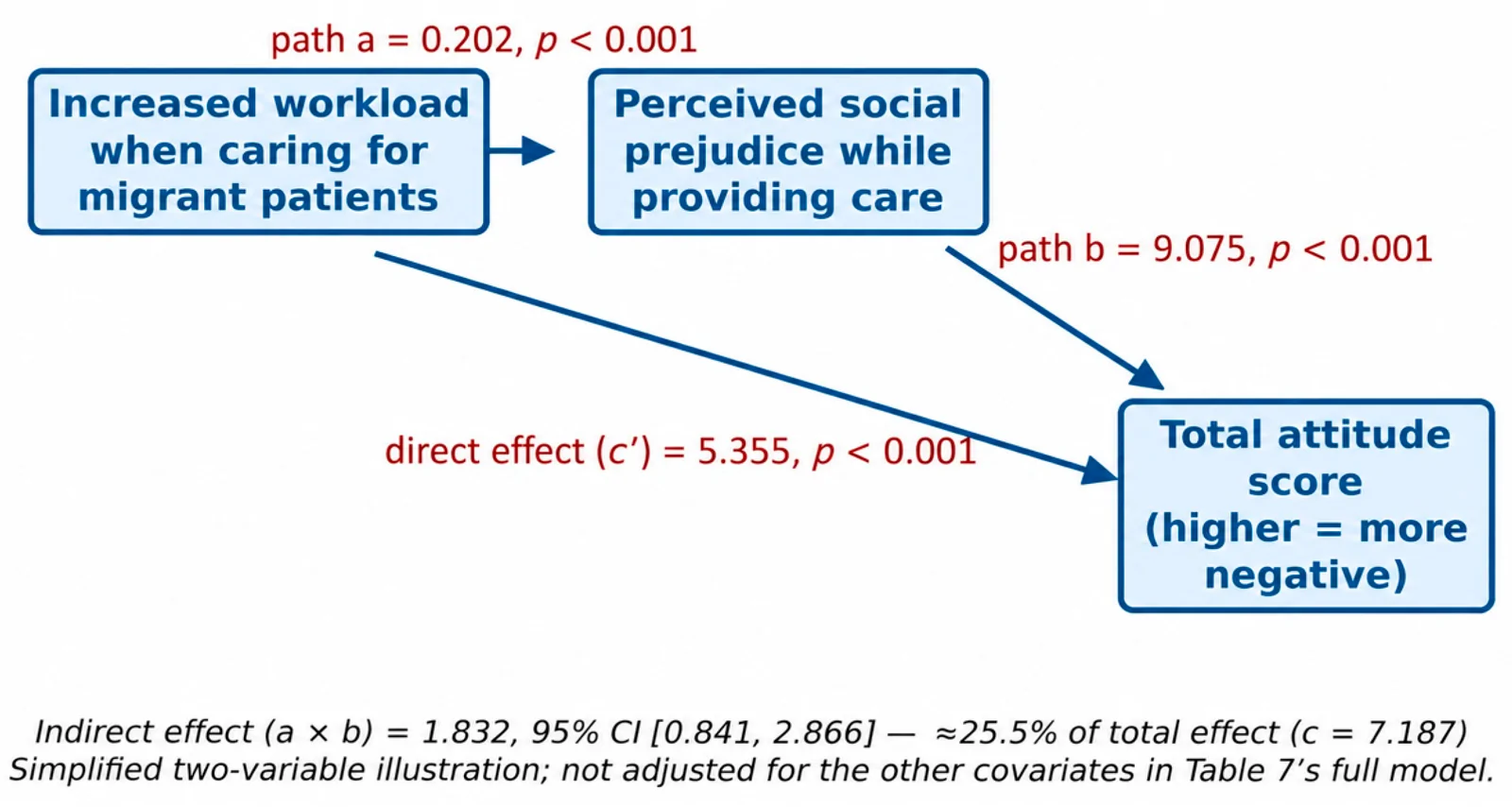
Illustrative pathway (mediation) diagram: workload → perceived social prejudice → attitude score.

To address whether the association between workload and attitudes is direct or operates, at least in part, indirectly through perceived social prejudice, an exploratory mediation Analysis was conducted using a linear product-of-coefficients approach (path a: workload → social prejudice; path b: social prejudice → total score, adjusting for workload; indirect effect = a × b, with bias-corrected 95% confidence intervals from 3000 bootstrap resamples). This simplified three-variable pathway model is presented as an illustrative, hypothesis-generating pathway diagram rather than a confirmatory causal test: because the data are cross-sectional, the temporal ordering of workload, perceived prejudice, and attitudes cannot be established, and unmeasured confounding cannot be excluded. The proposed directed pathway (Figure 2) reflects our conceptual model (workload → perceived prejudice → attitudes) rather than a data-driven or formally tested DAG.

Increased workload was significantly associated with a higher likelihood of perceiving social prejudice (path a = 0.202, *p* < 0.001), and perceived social prejudice was in turn significantly associated with a higher total attitude score, controlling for workload (path b = 9.075, *p* < 0.001). The total effect of workload on the total score was c = 7.187; this decomposed into a direct effect of c′ = 5.355 (*p* < 0.001) and an indirect effect through perceived social prejudice of a × b = 1.832 (bootstrap 95% CI 0.841–2.866), which did not include zero and therefore was consistent with a possible indirect statistical pathway, accounting for approximately 25.5% of the total association. In other words, the estimated direct-path coefficient was larger than the estimated indirect-path coefficient. Still, a meaningful minority operates indirectly through perceived social prejudice—consistent with the possibility that heavier workloads increase the perception of social prejudice while providing care, which in turn is associated with more negative attitudes. Given the cross-sectional design, this pathway should be interpreted as hypothesis-generating and tested prospectively in future longitudinal research.

## 4. Discussion

When the attitudes of healthcare professionals toward migrants are evaluated in light of the literature, a multidimensional pattern emerges. The fact that positive attitude scores in this study were not entirely low, while negative and inhibitory attitudes increased according to certain variables, is largely consistent with previous literature indicating that healthcare professionals’ attitudes toward migrants cannot be explained solely by individual characteristics; working conditions, communication difficulties, perceived workload, and institutional support also appear to play a role in these attitudes. It has been reported that linguistic, cultural, and institutional barriers also negatively affect migrants’ access to health services and the quality of care provided [20].

As noted in the literature, fostering positive attitudes toward migrant health can be achieved not only through individual awareness but also by strengthening communication processes, promoting cultural competence training, and developing institutional support mechanisms. Systematic reviews published in recent years show that language barriers, cultural differences, and inadequate interpreter support are associated with reduced access to health care for migrants and greater difficulties for health care providers in delivering care; conversely, cultural competence training, professional interpretation services, and institutional support initiatives are associated with more inclusive attitudes among health professionals [4,20,21,22]. In addition to in-person interpretation services and cultural competence training, digital solutions such as machine-assisted translation tools and remote interpretation via telehealth services have been proposed as scalable complements to in-person interpretation; however, significant ethical considerations regarding accuracy, confidentiality, and equitable access to these technologies for migrant patients with varying levels of digital literacy have also come to the fore [23].

The fact that married participants scored higher on measures of negative and inhibitory attitudes compared to single participants, and that those dissatisfied with their profession scored higher on negative attitudes and total scale scores, is consistent with the possibility that attitudes toward migrants may be related not only to perceptions of migrant patients but also to professional variables such as burnout, role strain, and job satisfaction; given the cross-sectional design, this remains an association rather than a proven explanation. The literature reports that health professionals’ conscious or unconscious biases may negatively affect patient-health professional interactions and the quality of care [24,25]. A recent report published by AHRQ also notes that healthcare professionals’ biases may contribute to unequal care provision for migrant patients and poorer health outcomes [24].

In this sample, it is noteworthy that as the number of migrant patients encountered in the past month increased, negative attitudes and total scale scores also tended to be higher. Although the literature shows that positive and structured interactions can reduce bias, interactions under heavy workload, time pressure, and communication difficulties may instead be associated with more negative experiences [20,26]. Similarly, a recent study conducted in Turkey reported that positive contact with migrants can reduce prejudice; however, without adequate institutional support, increased frequency of contact is associated with greater stress among healthcare professionals [27]. The fact that the vast majority of participants in our study perceived that providing care to migrant patients increased their workload is consistent with this interpretation; however, we emphasize that this is a relationship observed at a single point in time. One of the study’s more consistent findings is that language barriers, cultural differences, and interpreter support are associated with healthcare professionals’ attitudes toward migrants. The fact that healthcare professionals who reported language barriers and cultural differences had higher negative attitude scores suggests that communication difficulties may be more than just a technical issue; they may complicate the care process. Similarly, it was found that participants who believed that migrants’ attitudes toward health and illness made the provision of care more difficult had higher scores on the negative attitude and total scale measures. Previous studies have shown that cultural differences regarding health and illness can complicate the provision of care to migrants [10,28]. Similarly, a recent study conducted with nurses in Italy reported that differences in migrant patients’ health beliefs and expectations can create difficulties in the care process. These findings suggest that cultural understandings of health and illness may be related to healthcare professionals’ care experiences and attitudes [29].

A limited understanding of migrants’ cultural practices regarding illness, treatment, privacy, family involvement, and health-seeking behaviors may significantly contribute to misunderstandings during care. The literature also links language and cultural barriers to reduced access to health care, lower patient safety, poorer treatment adherence, and weaker trust between patients and providers; conversely, it associates culturally sensitive care practices and professional interpretation services with improvements in communication quality, patient satisfaction, and continuity of care [4,5,10,20,21,27,28,29,30]. These findings suggest that communication difficulties in migrant health cannot be explained solely by individual skills and that cultural competence, professional interpretation support, and institutional communication mechanisms may require attention. The fact that participants scored higher on negative and inhibitory attitude scales when they perceived attitudes toward migrants as negative or neutral, along with similar results among those who believed that social prejudice affects care, suggests that healthcare professionals’ attitudes may be related to both individual experience and the broader social and institutional context. Other studies have also reported that healthcare professionals’ conscious or unconscious biases weaken communication, undermine trust, and are associated with discriminatory care practices [23,31,32,33]. However, our cross-sectional data cannot directly confirm this mechanism; the observed pattern of relationships is consistent with it.

A notable finding of the study was that perceived social prejudice, increased workload, and reluctance to receive training on migrant health were independently associated with attitudes in both multivariate models, and these factors emerged as the most consistent findings. These findings are consistent with studies showing that healthcare professionals’ attitudes toward migrants are related not only to individual characteristics but also to broader contextual factors such as societal prejudice and working conditions [27,34]. Furthermore, studies reporting that receiving training on migrant health is associated with lower levels of xenophobia, whilst cross-cultural competence is associated with fewer negative attitudes, support our finding regarding the role of training [35,36].

The low proportion of participants who had received in-service training on migrant health, coupled with higher levels of negative and inhibitory attitudes among those unwilling to undergo such training, suggests that training could be a relevant avenue for fostering more positive attitudes; however, this interpretation should be made with caution. Notably, in this sample, having previously received training was not associated with attitude scores, and the desire to receive training in the future represents a stated intention rather than a measured outcome of training. Similarly, healthcare professionals who perceived themselves as less effective in providing care to migrant patients had higher negative and inhibitory attitude scores. However, because perceived effectiveness was assessed with a single subjective self-report item rather than a validated self-efficacy scale, this finding should be interpreted as an association with perceived effectiveness, not as evidence of professional self-efficacy. The broader literature suggests that cultural competence, communication skills, and knowledge of migrant health may support healthcare professionals’ confidence and communication with patients, and that cultural competence training may enhance care-related skills and sensitivity [4,6,19]. However, causal relationships between these factors and attitudes cannot be established from the present study.

### Limitations of the Study

This study has several limitations. First, the cross-sectional design precludes causal inference about the relationship between healthcare professionals’ characteristics and their attitudes toward migrant patients. Second, the inclusion of participants in the study through a Facebook-based professional community using convenience and snowball sampling methods increases the likelihood of selection bias and limits the generalizability of the findings. Furthermore, the fact that the sample consisted largely of women and nurses/midwives, along with the small sample sizes in some subgroups, makes it difficult to generalize the results to other healthcare professional groups. Third, the fact that the data were collected online and self-reported may have led to social desirability and common method biases. The fact that participants’ geographic locations were not specified also limits the assessment of the sample’s geographic representativeness. Although the Benjamini–Hochberg FDR correction was applied to reduce the likelihood of error arising from multiple comparisons, the findings must be interpreted with caution in light of these limitations. Nevertheless, the study provides comprehensive findings on healthcare professionals’ attitudes toward migrant patients by examining sociodemographic and occupational characteristics, experiences in providing care to migrants, communication barriers, and organizational factors, all within a relatively large sample. The results may contribute to future research and institutional strategies aimed at developing culturally sensitive and equitable health services for migrants.

## 5. Conclusions

In this cross-sectional study, healthcare professionals’ attitudes toward migrants were associated with communicative and organizational factors, as well as individual characteristics. Multivariate Analysis revealed that perceived societal prejudice, increased workload, and reluctance to receive training on migrant health were associated with both the total attitude score and negative attitudes; meanwhile, a lack of access to interpreters and the perception of migrants’ attitudes as negative were associated with the total attitude score.

These findings highlight the importance of professional interpreting services, cultural competence training, and organizational measures to reduce workload in migrant health services. However, due to the study’s cross-sectional design and reliance on convenience sampling, causal inferences cannot be drawn. Future longitudinal and experimental studies are recommended to evaluate these relationships and the effects of potential interventions.

## Figures and Tables

**Table 1:** Sociodemographic Characteristics of Participants.

Variable	Category	*n*	%/Median (Min–Max)
Age (years), mean ± SD: 31.33 ± 7.72; median (min–max): 29 (22–60)	<31 years	240	61.5
	≥31 years	150	38.5
Gender	Female	352	90.3
	Male	38	9.7
Marital status	Single	198	50.8
	Married	192	49.2
Education level	High school	20	5.1
	Associate’s degree	33	8.5
	Bachelor’s	267	68.5
	Graduate	70	17.9
Type of Healthcare Professional (Occupation)	Physician	7	1.8
	Nurse/Midwife	346	88.7
	Other	37	9.5
Years of professional experience	<1 year	68	17.4
	1–4 years	80	20.5
	5–10 years	99	25.4
	≥10 years	143	36.7
Voluntary choice of profession	Yes	196	50.3
	Partially	150	38.5
	No	44	11.3
Suitability of the profession	Yes	245	62.8
	Partially	111	28.5
	No	34	8.7
Job satisfaction	Yes	145	37.2
	Partially	167	42.8
	No	78	20.0

Data are presented as *n* (%) unless otherwise specified. Continuous variables are presented as mean ± SD and median (min–max). The “Other” healthcare professional category (*n* = 37) included paramedics (*n* = 10), emergency medical technicians/first and emergency aid technicians (*n* = 7), anesthesia technicians (*n* = 3), medical laboratory technicians (*n* = 3), an intern physician (*n* = 1), a radiology technician (*n* = 1), a medical secretary (*n* = 1), a health officer (*n* = 1), and other allied health professionals, each represented by one participant (*n* = 10 in total).

**Table 2:** Characteristics Related to the Care of Migrant Patients (*n* = 390).

Variable	Category	*n*	%
Number of migrant patients seen in the past month	≤3	153	39.2
	4–9	67	17.2
	10–15	71	18.2
	≥16	99	25.4
Most common migrant population	Newborns/Children	85	21.8
	Women	141	36.2
	Male	52	13.3
	Elderly	14	3.6
	All groups	98	25.1
The most common age group among migrants	0–5 years	80	20.5
	6–10 years	24	6.2
	11–15 years	2	0.5
	16–20 years	13	3.3
	21 years and older	271	69.5
The most common health service provided	Outpatient care/Emergency care	122	31.3
	Vaccination	13	3.3
	Prenatal and postnatal care	81	20.8
	Child health monitoring	31	7.9
	Reproductive health	3	0.8
	Services for sexually transmitted infections (STIs)	4	1.0
	All services	90	23.1
	Other	46	11.8
Encountering barriers while providing care to migrant patients	Yes	243	62.3
	Partially	103	26.4
	No	44	11.3
Types of barriers encountered (*n* = 346; among participants who reported any barriers) *	Language barrier	312	90.2
	Cultural differences	178	51.4
	Insufficient medical history	138	39.9
	Other	12	3.5
Availability of interpreters when communicating with migrant patients	Yes	209	53.6
	No	181	46.4
Perceived effectiveness in providing health care to migrant patients	Yes	139	35.6
	Partially	202	51.8
	No	49	12.6
Migrant patients’ perceived attitudes toward healthcare professionals	Positive	100	25.6
	Neutral	246	63.1
	Negative	44	11.3
The Perceived Impact of Social Prejudice While Providing Care	Yes	163	41.8
	No	227	58.2
The Impact of Migrants’ Attitudes Toward Health and Illness on Health Care Delivery	Facilitates	19	4.9
	No effect	146	37.4
	Makes it harder	225	57.7
Perceived change in workload when providing care to migrant patients	Decreased	1	0.3
	Remained the same	119	30.5
	Increased	270	69.2
Receiving in-service training on migrant health	Yes	13	3.3
	No	377	96.7
Desire to receive in-service training on migrant health	Yes	188	48.2
	No	202	51.8

Data are presented as *n* (%). Since participants could report more than one type of barrier, the sum of the percentages within a category may exceed 100%. * Multiple responses were allowed; therefore, percentages may exceed 100%.

**Table 3:** Descriptive Statistics, Reliability, and Distribution Characteristics of the Health Professionals’ Attitudes Toward Migrants Scale.

Scale	Observed Range	Mean ± SD	Cronbach’s α	Skewness	Kurtosis
Negative attitude	8–35	25.92 ± 7.39	0.890	−0.549	−0.710
Inhibitory Attitude	3–15	5.40 ± 3.06	0.802	1.533	1.858
Positive attitude	4–20	16.85 ± 3.48	0.780	−1.704	1.246
Total scale ^†^	16–68	38.47 ± 10.71	0.867	0.231	−0.130

Abbreviations: SD, standard deviation. Cronbach’s α values of ≥0.70 indicate acceptable internal consistency. ^†^ The total scale score was calculated by reverse-scoring the positive attitude items; therefore, higher total scores indicate more negative overall attitudes toward migrants.

**Table 4:** Comparison of Attitude Scale Scores According to Participants’ Sociodemographic Characteristics. (Marital status and job satisfaction: primary/confirmatory comparison—included in the multivariate model. All other variables: secondary/exploratory comparison).

Variable	Category	Negative Attitude Mean ± SD	Inhibitory Attitude Mean ± SD	Positive Attitude Mean ± SD	Total Score Mean ± SD
Age	<31 years	25.99 ± 7.10	5.30 ± 3.10	17.10 ± 3.34	38.18 ± 10.86
	≥31 years	25.81 ± 7.85	5.57 ± 3.01	16.44 ± 3.68	38.94 ± 10.49
	t/U	0.235	−0.870	1.826	−0.678
	*p*	0.814	0.385	0.069	0.498
Gender	Female	26.01 ± 7.31	5.34 ± 2.97	16.95 ± 3.31	38.40 ± 10.27
	Male	25.03 ± 8.11	5.97 ± 3.85	15.87 ± 4.75	39.13 ± 14.34
	t/U	0.783	−1.210	1.827	−0.398
	*p*	0.434	0.227	0.069	0.691
Marital status	Single	24.92 ± 7.37	5.04 ± 2.90	16.98 ± 3.50	36.97 ± 10.92
	Married	26.94 ± 7.28	5.78 ± 3.19	16.70 ± 3.47	40.02 ± 10.29
	t/U	−2.720	−2.418	0.798	−2.833
	*p*	**0.007**	**0.016**	0.425	**0.005**
Educational level	High school ^1^	28.80 ± 7.37	7.25 ± 4.41	16.40 ± 4.72	43.65 ± 11.72
	Associate’s degree ^2^	26.85 ± 7.28	5.70 ± 3.08	16.48 ± 3.52	40.06 ± 10.18
	Bachelor’s degree ^3^	25.76 ± 7.36	5.34 ± 3.08	16.78 ± 3.50	38.33 ± 10.82
	Graduate ^4^	25.24 ± 7.48	4.97 ± 2.30	17.41 ± 2.99	36.80 ± 9.88
	F/H	1427	3072	0.884	2411
	*p*	0.234	**0.028** (post hoc: 1 > 3, 1 > 4)	0.449	0.067
Occupation	Physician (*n* = 7) *	25.43 ± 9.71	6.29 ± 4.42	17.86 ± 2.97	37.86 ± 14.98
	Nurse/Midwife	25.88 ± 7.36	5.41 ± 3.08	16.83 ± 3.57	38.47 ± 10.69
	Other	26.32 ± 7.38	5.14 ± 2.63	16.84 ± 2.72	38.62 ± 10.33
	F/H	0.075	0.433	0.299	0.015
	*p*	0.928	0.649	0.742	0.985
Years of professional experience	<1 year	24.75 ± 6.76	4.84 ± 2.71	17.04 ± 3.06	36.54 ± 9.77
	1–4 years	24.99 ± 7.50	5.33 ± 3.25	16.81 ± 3.95	37.50 ± 11.79
	5–10 years	27.29 ± 6.58	5.86 ± 3.39	17.00 ± 3.30	40.15 ± 10.75
	≥10 years	26.04 ± 8.02	5.40 ± 2.86	16.66 ± 3.55	38.78 ± 10.38
	F/H	2.166	1.523	0.268	1.815
	*p*	0.092	0.208	0.848	0.144
Voluntary choice of profession	Yes	25.62 ± 7.68	5.59 ± 3.15	16.62 ± 3.87	38.59 ± 11.22
	Partially	26.33 ± 7.15	5.25 ± 2.92	17.07 ± 3.08	38.52 ± 10.26
	No	25.82 ± 6.96	5.09 ± 3.17	17.11 ± 2.93	37.80 ± 10.10
	F/H	0.397	0.759	0.852	0.101
	*p*	0.673	0.469	0.427	0.904
Suitability of the profession	Yes	25.38 ± 7.47	5.24 ± 2.99	16.98 ± 3.58	37.64 ± 10.63
	Partially	26.57 ± 7.11	5.47 ± 3.00	16.77 ± 3.28	39.27 ± 10.57
	No	27.71 ± 7.44	6.35 ± 3.68	16.15 ± 3.46	41.91 ± 11.16
	F/H	2097	2013	0.893	2833
	*p*	0.124	0.135	0.410	0.060
Job satisfaction	Yes ^1^	24.90 ± 7.69	5.18 ± 2.99	17.10 ± 3.61	36.99 ± 11.05
	Partially ^3^	25.90 ± 7.16	5.38 ± 3.04	16.83 ± 3.29	38.45 ± 10.28
	No ^2^	27.83 ± 7.00	5.87 ± 3.23	16.41 ± 3.65	41.29 ± 10.54
	F/H	4.052	1.307	0.986	4.172
	*p*	**0.018** (post hoc: 2 > 1)	0.272	0.374	**0.016** (post hoc: 2 > 1)

Superscript numbers indicate the educational level categories used in post hoc comparisons: ^1^ High school; ^2^ Associate’s degree; ^3^ Bachelor’s degree; ^4^ Graduate. * Indicates the category with the smallest sample size. Data are presented as mean ± SD. For comparisons between two groups, the independent samples *t*-test or the Mann–Whitney U test was used; for comparisons involving three or more groups, one-way ANOVA (F) or the Kruskal–Wallis (H) test was used. Data are presented as mean ± SD. Where appropriate, an independent samples *t*-test, one-way ANOVA, Mann–Whitney U test, or Kruskal–Wallis test was used. A Bonferroni post hoc analysis was applied following overall comparisons. Statistically significant *p*-values (*p* ≤ 0.001) are shown in bold.

**Table 6:** Comparison of Attitude Scale Scores According to Barriers, Perceptions, Workload, and Training Related to the Care of Migrant Patients.

Variable	Category	Negative Attitude Mean ± SD	Inhibitory Attitude Mean ± SD	Positive Attitude Mean ± SD	Total Score Mean ± SD
Encountering obstacles while providing care	Yes (1)	26.95 ± 7.08	5.62 ± 3.17	16.74 ± 3.59	39.84 ± 10.42
	No (2)	21.73 ± 8.89	4.45 ± 2.72	17.02 ± 3.85	33.16 ± 11.73
	Partially (3)	25.26 ± 6.74	5.29 ± 2.90	17.03 ± 3.07	37.52 ± 10.21
	F/H	10.354	2.820	0.318	8.081
	*p*	<0.001 ** (post hoc: 1 > 2, 3 > 2)	0.061	0.728	<0.001 ** (post hoc: 1 > 2)
Language barrier	Yes	26.36 ± 7.01	5.34 ± 2.95	16.96 ± 3.18	38.75 ± 10.26
	No	24.14 ± 8.57	5.65 ± 3.49	16.41 ± 4.50	37.38 ± 12.36
	t/U	2.389	−0.809	1.236	1.005
	*p*	**0.017**	0.419	0.217	0.316
Cultural differences	Yes	27.38 ± 6.64	5.67 ± 3.21	16.79 ± 3.38	40.26 ± 10.10
	No	24.69 ± 7.76	5.18 ± 2.93	16.90 ± 3.57	36.97 ± 11.00
	t/U	3.642	1.574	−0.309	3.056
	*p*	<0.001 **	0.116	0.757	**0.002**
Insufficient medical history	Yes	26.60 ± 6.81	5.46 ± 2.97	16.80 ± 3.21	39.26 ± 10.29
	No	25.54 ± 7.67	5.37 ± 3.12	16.87 ± 3.63	38.04 ± 10.93
	t/U	1.354	0.292	−0.175	1.073
	*p*	0.177	0.771	0.861	0.284
Availability of an interpreter when needed	Yes	25.39 ± 7.39	4.85 ± 2.52	17.40 ± 2.83	36.85 ± 9.92
	No	26.52 ± 7.36	6.04 ± 3.50	16.21 ± 4.03	40.35 ± 11.29
	t/U	−1.512	−3.884	3.402	−3.264
	*p*	0.131	<0.001 **	0.001 **	0.001 **
Perceived effectiveness in providing care	Yes (1)	24.39 ± 7.75	4.99 ± 2.98	17.33 ± 3.43	36.04 ± 10.58
	No (2)	28.37 ± 6.66	6.41 ± 3.31	15.43 ± 4.08	43.35 ± 10.88
	Partially (3)	26.38 ± 7.11	5.45 ± 3.01	16.86 ± 3.28	38.97 ± 10.33
	F/H	6.224	4.007	5.530	9.240
	*p*	**0.002** (post hoc: 2 > 1, 3 > 1)	**0.019** (post hoc: 2 > 1)	**0.004** (post hoc: 1 > 2)	<0.001 ** (post hoc: 2 > 1, 2 > 3, 3 > 1)
Migrant patients’ perceived attitudes toward healthcare professionals	Positive (1)	22.72 ± 7.76	4.57 ± 2.75	17.86 ± 2.89	33.43 ± 10.59
	Neutral (2)	26.36 ± 6.84	5.59 ± 3.04	16.63 ± 3.46	39.33 ± 9.71
	Negative (3)	30.70 ± 6.36	6.23 ± 3.50	15.77 ± 4.30	45.16 ± 11.52
	F/H	21.010	5.909	7.026	22.729
	*p*	<0.001 ** (post hoc: 3 > 1, 3 > 2, 2 > 1)	**0.003** (post hoc: 2 > 1, 3 > 1)	0.001 ** (post hoc: 1 > 2, 1 > 3)	<0.001 ** (post hoc: 3 > 1, 3 > 2, 2 > 1)
The perceived effect of social bias while providing care	Yes	29.45 ± 5.70	6.65 ± 3.37	15.78 ± 3.57	44.33 ± 9.63
	No	23.38 ± 7.43	4.51 ± 2.47	17.61 ± 3.21	34.27 ± 9.41
	t/U	8.754	7.253	−5.301	10.303
	*p*	<0.001 **	<0.001 **	<0.001 **	<0.001 **
The Effect of Migrants’ Health/Illness Attitudes on Health Care Delivery	Facilitates (1)	22.37 ± 7.93	5.16 ± 3.83	16.16 ± 4.68	35.37 ± 13.68
	No effect (2)	23.92 ± 7.68	4.99 ± 2.71	17.10 ± 3.29	35.82 ± 10.71
	Makes it harder (3)	27.52 ± 6.73	5.69 ± 3.19	16.74 ± 3.49	40.46 ± 10.03
	F/H	13.638	2.363	0.845	9.579
	*p*	<0.001 ** (post hoc: 3 > 1, 3 > 2)	0.096	0.430	<0.001 ** (post hoc: 3 > 2)
Change in perceived workload	Decreased (*n* = 1) ^†^	8.00	3.00	18.00	17.00
	No change	22.30 ± 7.15	4.71 ± 2.56	17.42 ± 2.92	33.60 ± 9.71
	Increased	27.58 ± 6.84	5.71 ± 3.22	16.59 ± 3.69	40.70 ± 10.36
	t (No Change vs. Increased) ^†^	−6.981	−3.044	2.155	−6.405
	*p*	<0.001 **	**0.003**	**0.032**	<0.001 **
Receiving in-service training on migrant health	Yes	25.62 ± 8.39	5.62 ± 3.36	17.23 ± 3.27	38.00 ± 12.88
	No	25.93 ± 7.36	5.40 ± 3.06	16.83 ± 3.49	38.49 ± 10.65
	t/U	−0.150	0.254	0.404	−0.162
	*p*	0.881	0.799	0.686	0.871
Desire to receive in-service training on migrant health	Yes	23.16 ± 6.79	4.37 ± 2.37	17.69 ± 3.04	33.85 ± 8.93
	No	28.48 ± 7.00	6.36 ± 3.32	16.06 ± 3.68	42.78 ± 10.45
	t/U	−7.600	−6.765	4.749	−9.049
	*p*	<0.001 **	<0.001 **	<0.001 **	<0.001 **

Data are presented as mean ± SD. Where appropriate, an independent samples *t*-test, one-way ANOVA, Mann–Whitney U test, or Kruskal–Wallis test was used. A Bonferroni post hoc analysis was applied following overall comparisons. Statistically significant *p*-values (*p* ≤ 0.001) are shown in bold; *p*-values marked with ** also remained significant at the stricter α = 0.001 threshold applied, given the large number of repeated comparisons (see Section 3.1). ^†^ The “Decreased workload” category was excluded from the comparative analysis because it included only one participant (*n* = 1); therefore, the analysis was performed between the “No change” and “Increased” categories.

**Table 7:** Multivariate Linear Regression Model for the Total Attitude Score (Primary Outcome; *n* = 384).

Predictor	B (Unstandardized)	95% CI	Standardized β	*p*
**Constant (Cutoff point)**	**25.554**	22.667–28.441	**—**	**<0.001**
Marital status: Married (ref: Single)	1023	−0.753–2.799	0.048	0.259
Job dissatisfaction (ordinal: Yes = 0, Partially = 1, No = 2)	0.152	−1.080–1.385	0.010	0.808
Reporting a language barrier (ref: Did not report)	−0.913	−3.303–1.478	−0.034	0.454
Reporting cultural differences (ref: Those who did not report)	1.268	−0.452–2.989	0.059	0.149
Inaccessibility of an interpreter (ref: Accessible)	2.378	0.577–4.180	0.111	0.010
Perceived low effectiveness (ordinal: Yes = 0, Partially = 1, No = 2)	1.204	−0.231–2.638	0.074	0.100
Negative perception of migrants’ attitudes (ordinal: Positive = 0, Neutral = 1, Negative = 2)	1.919	0.192–3.646	0.106	0.029
Perception of social prejudice (ref: Those who do not perceive it)	**6.500**	4.705–8.295	**0.300**	**<0.001**
Increase in workload (ref: No change; *n* = 1—the “decreased” category has been excluded)	**4.215**	2.154–6.277	**0.181**	**<0.001**
Reluctance to pursue education (ref: Willing)	**6149**	4.311–7.988	**0.287**	**<0.001**

B: unstandardized regression coefficient; CI: confidence interval; β: standardized regression coefficient. Calculated using Robust (HC3) standard errors. R^2^ = 0.398; adjusted R^2^ = 0.382; F(10, 373) = 23.28; *p* < 0.001. All VIF values < 1.25. Statistically significant predictors (*p* ≤ 0.001) are shown in bold.

**Table 8:** Multivariate Linear Regression Model for the Negative Attitude Subscale (Sensitivity Analysis; *n* = 384).

Predictor	B	95% CI	β	*p*
**Constant (Cutoff point)**	**17.037**	14,818–19,255	**—**	**<0.001**
Marital status: Married (ref: Single)	0.691	−0.579–1.961	0.047	0.286
Job dissatisfaction (ordinal)	0.082	−0.798–0.963	0.008	0.855
Reporting a language barrier	0.573	−1.187–2.332	0.031	0.523
Reporting cultural differences	1.205	−0.037–2.447	0.082	0.057
Inaccessibility of an interpreter	0.620	−0.672–1.911	0.042	0.347
Perceived low effectiveness (ordinal)	0.456	−0.547–1.459	0.041	0.373
Negative perception of migrants’ attitudes (ordinal)	1.174	−0.026–2.374	0.094	0.055
**Perception of social prejudice**	**3.715**	2.384–5.047	**0.250**	**<0.001**
**Increase in Workload**	**3432**	1939–4925	**0.216**	**<0.001**
**Reluctance to undergo training**	**3659**	2.305–5.013	**0.249**	**<0.001**

B: unstandardized regression coefficient; CI: confidence interval; β: standardized regression coefficient. Calculated using Robust (HC3) standard errors. R^2^ = 0.327; adjusted R^2^ = 0.309; F(10, 373) = 20.81; *p* < 0.001. Statistically significant predictors (*p* ≤ 0.001) are shown in bold.

## Data Availability

The datasets generated and/or analyzed during the current study are not publicly available due to ethical and confidentiality constraints; however, they can be obtained from the corresponding author upon reasonable request.

## Supplementary Materials

The following supporting information can be downloaded at: https://www.mdpi.com/article/10.3390/healthcare14182984/s1, Table S1: Complete Statistical Breakdown of All Pairwise and Multi-Group Comparisons.
