# Supplementary material for: Factors Associated with Healthcare Professionals’ Attitudes Toward Migrant Patients: A Cross-Sectional, Exploratory Study
Source: Healthcare (Basel). 2026 Sep 12;14(18):2984. doi: 10.3390/healthcare14182984 (PMC13606382; doi:10.3390/healthcare14182984)
Supplement: Supplementary file 1 [file healthcare-14-02984-s001.zip › healthcare-4481191-supplementary.pdf]

Supplementary Material: STROBE Statement — Checklist of Items That Should Be Included in Reports of Cross-Sectional Studies

Manuscript: "Factors Associated with Healthcare Professionals' Attitudes Toward Migrant Patients: A Cross-Sectional, Exploratory Study"

Table S1. Complete Statistical Breakdown of All Pairwise and Multi-Group Comparisons.

**Section/Topic**	**Item No**	**Recommendation**	**Reported on page/section**
Title and abstract	1(a)	Indicate the study's design with a commonly used term in the title or the abstract.	Title ("Cross-Sectional, Exploratory Study"); Abstract, Methods sentence.
	1(b)	Provide in the abstract an informative and balanced summary of what was done and what was found.	Abstract.
Introduction	2	Explain the scientific background and rationale for the investigation being reported.	Section 1, Introduction, paragraphs 1–4.
	3	State specific objectives, including any prespecified hypotheses.	Section 1, Introduction, final paragraph (aim of the study).
Methods	4	Present key elements of study design early in the paper.	Section 2.1, first paragraph.
	5	Describe the setting, locations, and relevant dates, including periods of recruitment, exposure, and data collection.	Section 2.1–2.2 (recruitment via Facebook Reproductive Health Volunteers Community; data collection March 2025–March 2026).
	6(a)	Give the eligibility criteria, and the sources and methods of selection of participants.	Section 2.1 (eligibility, inclusion/exclusion criteria) and Section 2.2 (recruitment/snowball sampling).

	7	Clearly define all outcomes, exposures, predictors, potential confounders, and effect modifiers; give diagnostic criteria if applicable.	Section 2.2 (Individual Assessment Form; Health Professionals' Attitudes Toward Migrants Scale, subscale/total scoring).
	8*	For each variable of interest, give sources of data and details of methods of assessment. Describe comparability of assessment methods if more than one group.	Section 2.2 (self-report questionnaire, Google Forms; single assessment method for all participants).
	9	Describe any efforts to address potential sources of bias.	Section 2.2 (email verification/one response per account) and Section 2.5, Potential Sources of Bias.
	10	Explain how the study size was arrived at.	Section 2.1, sample size paragraph (finite-population and a priori power calculations).
	11	Explain how quantitative variables were handled in the analyses; describe groupings chosen and why.	Section 2.3, Data Analysis (scale scoring; categorical groupings in Tables 1–2).
	12(a)	Describe all statistical methods, including those used to control for confounding.	Section 2.3, Data Analysis and Multivariable analysis paragraph.
	12(b)	Describe any methods used to examine subgroups and interactions.	Section 2.3, "Distinction between primary and exploratory analyses" and "Handling of small subgroups" subsections.
	12(c)	Explain how missing data were addressed.	Section 2.2 (all items mandatory, minimizing missing data) and Section 3.2/3.3 (listwise

			deletion, n reported for each regression model).
	12(d)	If applicable, describe analytical methods taking account of sampling strategy.	Not applicable — non-probability convenience/snowball sample; addressed as a limitation in Section 2.5 and Discussion.
	12(e)	Describe any sensitivity analyses.	Section 3.2 (negative-subscale sensitivity model, Table 8) and Section 3.3 (occupation-adjusted sensitivity model, Table 9).
Results	13(a)	Report numbers of individuals at each stage of the study.	Figure 1, participant flow diagram; Section 3, first paragraph.
	13(b)	Give reasons for non-participation at each stage.	Section 2.2 / Section 2.5 (number of non-completers not available; response rate could not be calculated — stated as a limitation).
	13(c)	Consider use of a flow diagram.	Figure 1.
	14(a)	Give characteristics of study participants and information on exposures and potential confounders.	Tables 1 and 2; Section 3, descriptive paragraphs.
	14(b)	Indicate number of participants with missing data for each variable of interest.	Section 2.2 (no item-level missing data, as all items were mandatory); n reported per analysis in Tables 7–9.
Outcome data	15*	Report numbers of outcome events or summary measures.	Table 3 (descriptive statistics of the attitude scale) and Section 3, paragraph preceding Table 3.
Main results	16(a)	Give unadjusted and confounder-adjusted estimates and their precision (e.g., 95%	Tables 4–6 (unadjusted); Tables 7–9 (adjusted, with 95% CI).

		CI); state which confounders were adjusted for.	
	16(b)	Report category boundaries when continuous variables were categorized.	Tables 1–2 and their footnotes/category definitions.
	16(c)	If relevant, consider translating relative risk estimates into absolute risk.	Not applicable — outcome is a continuous attitude score, not a risk/odds outcome.
	17	Report other analyses done — e.g., subgroup analyses, sensitivity analyses.	Section 3.1 (effect sizes, FDR and α = 0.001 correction), Section 3.3 (Table 9), Section 3.4 (mediation analysis, Figure 2).
Discussion	18	Summarise key results with reference to study objectives.	Section 4, first paragraphs.
	19	Discuss limitations, taking into account sources of potential bias or imprecision, and their direction/magnitude.	Section 2.5, Potential Sources of Bias, and Limitations subsection in Section 4.
	20	Give a cautious overall interpretation considering objectives, limitations, multiplicity of analyses, and other evidence.	Section 4, Discussion and Conclusion.
	21	Discuss the generalisability (external validity) of the study results.	Section 2.5 and Section 4 (predominantly nurse/midwife and female sample; convenience/snowball sampling).
Other information	22	Give the source of funding and the role of funders.	Funding statement, end of manuscript.

**Given the study is descriptive in nature, item 8 refers to the assessment of variables/scale scores rather than exposure ascertainment; item 15 refers to the summary attitude-score statistics rather than clinical outcome events.*

This checklist is based on: von Elm E, Altman DG, Egger M, Pocock SJ, Gøtzsche PC, Vandenbroucke JP; STROBE Initiative. The Strengthening the Reporting of Observational Studies in Epidemiology (STROBE) statement: guidelines for reporting observational studies. Lancet. 2007;370(9596):1453-1457.
